# Intratendinous Injection of Hyaluronate Induces Acute Inflammation: A Possible Detrimental Effect

**DOI:** 10.1371/journal.pone.0155424

**Published:** 2016-05-13

**Authors:** Po-Ting Wu, I-Ming Jou, Li-Chieh Kuo, Fong-Chin Su

**Affiliations:** 1 Institute of Biomedical Engineering, National Cheng Kung University, Tainan, Taiwan; 2 Department of Orthopedics, National Cheng Kung University Hospital, Tainan, Taiwan; 3 Department of Occupational Therapy, National Cheng Kung University, Tainan, Taiwan; 4 Medical Device Innovation Center, National Cheng Kung University, Tainan, Taiwan; Mayo Clinic Minnesota, UNITED STATES

## Abstract

Hyaluronate (HA) is therapeutic for tendinopathy, but an intratendinous HA injection is usually painful; thus, it is not suggested for clinical practice. However, there are no studies on the histopathological changes after an intratendinous HA injection. We hypothesized that an HA injection would induce more-acute inflammation than that induced by an injection of phosphate buffered saline (PBS). Thirty-two rats were randomly divided into 4 post-injection groups (n = 8): *day 3*, *day 7*, *day 28*, *and day 42*. HA (0.1 c.c.) was, using ultrasound guidance, intratendinously injected into each left Achilles tendon, and PBS (0.1 c.c.) into each right one. For each group, both Achilles tendons of 3 control-group rats (n = 6) were given only needle punctures. The histopathological score, ED1+ and ED2+ macrophage densities, interleukin (IL)-1β expression, and the extent of neovascularization were evaluated. In both experimental groups, each Achilles tendon showed significant histopathological changes and inflammation compatible with acute tendon injury until *day 42*. The HA group showed more-significant (p < 0.05) histopathological changes, higher ED1+ and ED2+ macrophage density, and higher IL-1β expression than did the PBS group. The neovascularization area was also significantly (p < 0.05) greater in the HA group, except on day 3. Both HA and PBS induced acute tendon injury and inflammation, sequential histopathological changes, ED1^+^ and ED2^+^ macrophage accumulation, IL-1β expression, and neovascularization until post-injection *day 42*.HA induced more-severe injury than did PBS. Therefore, an intratendinous HA injection should be avoided.

## Introduction

Tendinopathy is a chronic painful tendon disorder that is prevalent in the athletic and the sedentary. Tendon overuse injury has been claimed to account for 30–50% of all sports-related injuries[1] and almost half of all occupational illnesses in the United States [2]. Nonsurgical management is generally accepted as the first choice of treatment for most cases[3–5]. A corticosteroid injection is one of the most common treatments[5]. However, it yields only short-term benefits[5, 6] and might cause adverse effects, including tendon ruptures[7, 8]. These complications have contributed to the greater use of other locally injectable therapeutics, such as lauromacrogol (polidocanol), platelet-rich plasma, botulinum toxin, proteinases, and hyaluronate (HA). There is growing evidence from clinical trials[6, 9–12] and animal studies[13–15] that HA effectively mitigates tendinopathy. Even though the true mechanism of HA on tendinopathy remains unclear, its effects might result from anti-inflammation in interleukin (IL)-1-stimulated subacromial synovial fibroblasts[16]and partial restoration of tendon structures in various rat tendinopathy models [13–15].

Because an intratendinous injection of HA usually leads to sharp pain, treating tendinopathy with a non-intratendinous injection is clinically preferred[9, 11, 12, 17, 18]. Furthermore, an intratendinous injection might weaken the tendon structure and increase the probability that the tendon will rupture[6]. Therefore, a paratendinous injection of HA is anecdotally favored[11, 18, 19] despite a lack of evidence that a direct intratendinous injection leads to an acute injury.

After an acute tendon injury, different subtypes of leukocytes are rapidly recruited by the inflamed tissue to phagocytose cell debris and stimulate fibroblast proliferation and collagen deposition[20]. In the acute tendon inflammation model induced by a collagenase injection, the macrophage (MΦ) is the predominant type of leukocyte from post-injection *day 3* until the inflammation ends[21]. The infiltrated MΦs will upregulate proinflammatory factors, especially IL-1β[22]. Increased angiogenesis and MΦ infiltration are hallmarks of acute tendon injury and essential for sequential tendon repair[23]. Therefore, we evaluated the histopathological change and inflammatory responses after an intratendinous injection of HA in a rat model. We hypothesized that the HA injection would induce acute inflammation that showed worse histopathological results, more MΦ infiltration, higher IL-1β expression, and more angiogenesis than induced by an injection of phosphate buffered saline (PBS).

## Materials and Methods

### Ethics statement

All of the experimental rats were purchased from the Animal Center at National Cheng Kung University, and the following experiments were done in accordance with protocols approved by the institutional Animal Care and Use Committee of National Cheng Kung University (protocol number: IACUC-102064).All of the rats were housed in the Animal center at a temperature of 25em±2emperature of the Cheng Kung Unprovided. All surgery and imaging was done after the rats had been anesthetized with isofluorane, and every effort was made to minimize their suffering. All of the rats were humanely euthanized using carbon dioxide, as prescribed in an IACUC-approved animal research protocol.

### Animal model

Thirty-two male Sprague-Dawley rats (350–400 g) were randomly assigned to one of 4 post-injection groups (n = 8): *day 3*, *day 7*, *day 28*, and *day 42*. Each rat was intratendinously injected with HA in the left Achilles tendon and with PBS in the right one. Equivalent doses (0.1 c.c.)[13] of 1% commercial HA (Arti-aid; Maxigen Biotech, New Taipei City, Taiwan) and sterile PBS containing 150 mMNaCl and 50 mM NaH_2_PO4 [pH 7.4] was injected into the left and right Achilles tendons with a 29G needle. Both Achilles tendons of twelve control-group rats were given needle puncture only and randomly assigned to one of 4 time-points (n = 6 tendons). Every injection and needle puncture procedure was guided by using an ultrasound (US) (Vevo 770; VisualSonics, Toronto, Canada) 55-MHz linear transducer for high-resolution images. After the index procedure, the rats were allowed normal activity in their cages. The experimental rats were euthanized on the post-injection days indicated in their group names: *days 3*, *7*, *28*, and *42*.

### Ultrasonographic guidance procedures

**Fig 1:**
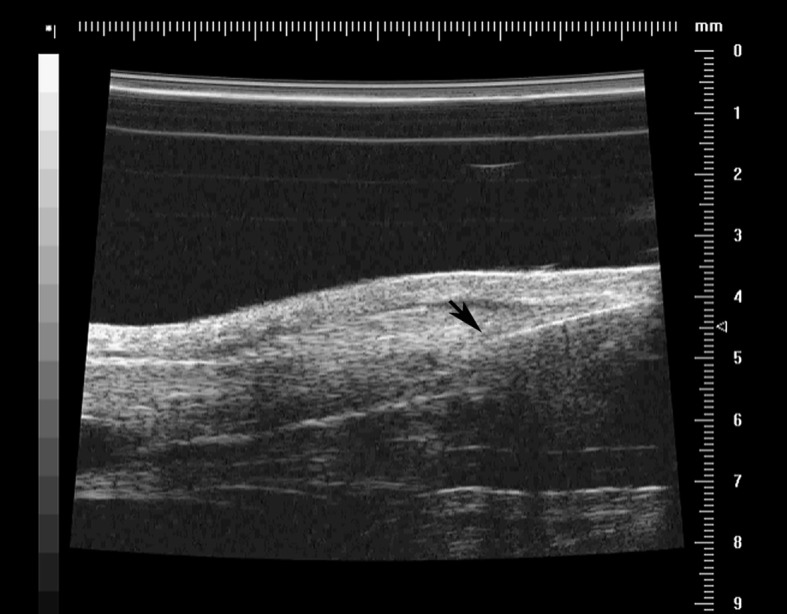
Ultrasound (US)-guided intratendinous injection. The needle (arrow) is clearly visualized with US guidance. The image of the tendon tissue expansion confirms the effect of the high-volume intratendinous injection.

Before the US examination, the posterior skin hair of both hind limbs was removed using an electric razor. The rat was placed prone on a flat, heated pad to maintain its body temperature. The hind limbs were secured, and a sterile coupling gel was loaded to cover the Achilles tendon after it had been disinfected. Two-dimensional real-time B-mode scanning was used to visualize the Achilles tendon, and the scanning window was centered on the tendon, with a 10.0 × 10.0-mm field of view. The ultrasound transducer was held in a hand-free stand positioned 4.5 mm above the center of the tendon. The axis of the linear transducer was aligned along the long axis of the Achilles tendon. Using real-time US guidance, the needle was placed parallel to the long axis of the US transducer and the injection was confirmed to be intratendinous (Fig 1). The US images clearly showed the slow inflation of the tendon as it was infiltrated by HA or PBS.

### Histopathological grade

Both Achilles tendons in each rat were harvested from the muscle-tendon junction to the calcaneal insertion site. Each specimen was fixed in fresh 4% paraformaldehyde for 16–24 h at 4°C, and then subsequently dehydrated, paraffin-embedded, and longitudinally sectioned. Sequential 4-μM sections were stained with hematoxylin and eosin (H&E). We used a semiquantitative method to score each factor on a 4-point scale scoring system: 0 = normal, 1 = slightly abnormal, 2 = moderately abnormal, and 3 = markedly abnormal[18]. The following parameters were assessed: fiber structure, fiber arrangement, roundness of the nuclei, regional variations in cellularity, increased vascularity, decreased collagen stainability, fibrosis or hyalinization, and presence of acute inflammation characteristics. The maximum total score for each specimen was 24. Using a light microscope, two blinded examiners assessed the extent of tendinosis. The kappa (κ) value was used for interobserver reliability. If the two examiners gave different scores, the discrepancy was resolved by mutual agreement after a discussion.

### Immunohistochemistry

ED1^+^ and ED2^+^ MΦs, IL-1β, and blood vessels were labeled using the following antibodies: rabbit anti-ED1^+^ or rabbit anti-ED2^+^ MΦ (Serotec, Raleigh, NC), mouse anti-IL-1β (Millipore, Billerica, MA), and mouse anti-PECAM-1 (BD Biosciences Pharmingen, San Diego, CA). All sections were then washed three times with PBS for 10 min each and incubated with HRP-conjugated secondary antibody (Jackson ImmunoResearch Laboratories, West Grove, PA) for 1 h. The representative regions of interest were identified and examined, and high-resolution images were extracted from whole-tissue scans (TissueFAXS, Vienna, Australia). ED1^+^ and ED2^+^ MΦs were manually counted, and cell concentrations were calculated based on section area and thickness[23]. IL-1β^+^ cells were also manually counted and then expressed as a percentage of the total number of cells[24]. The surface areas occupied by blood vessels in the endotendon regions of the Achilles tendons were respectively quantified and expressed as percentages of the total areas[25], using ImageJ 1.46(http://imagej.nih.gov/ij/).

### Statistical analysis

All quantitative data are expressed as mean ± standard deviation (SD). Comparisons of histopathological grade, the concentration of MΦs, the expression of IL-1β, and the density of blood vessels in all groups were assessed using the Kruskal-Wallis test and the post-hoc Mann-Whitney U test. Significance was set at *p*< 0.05. Data were analyzed using SPSS 16.0 for Windows.

## Results

### Histopathological grade

**Fig 2:**
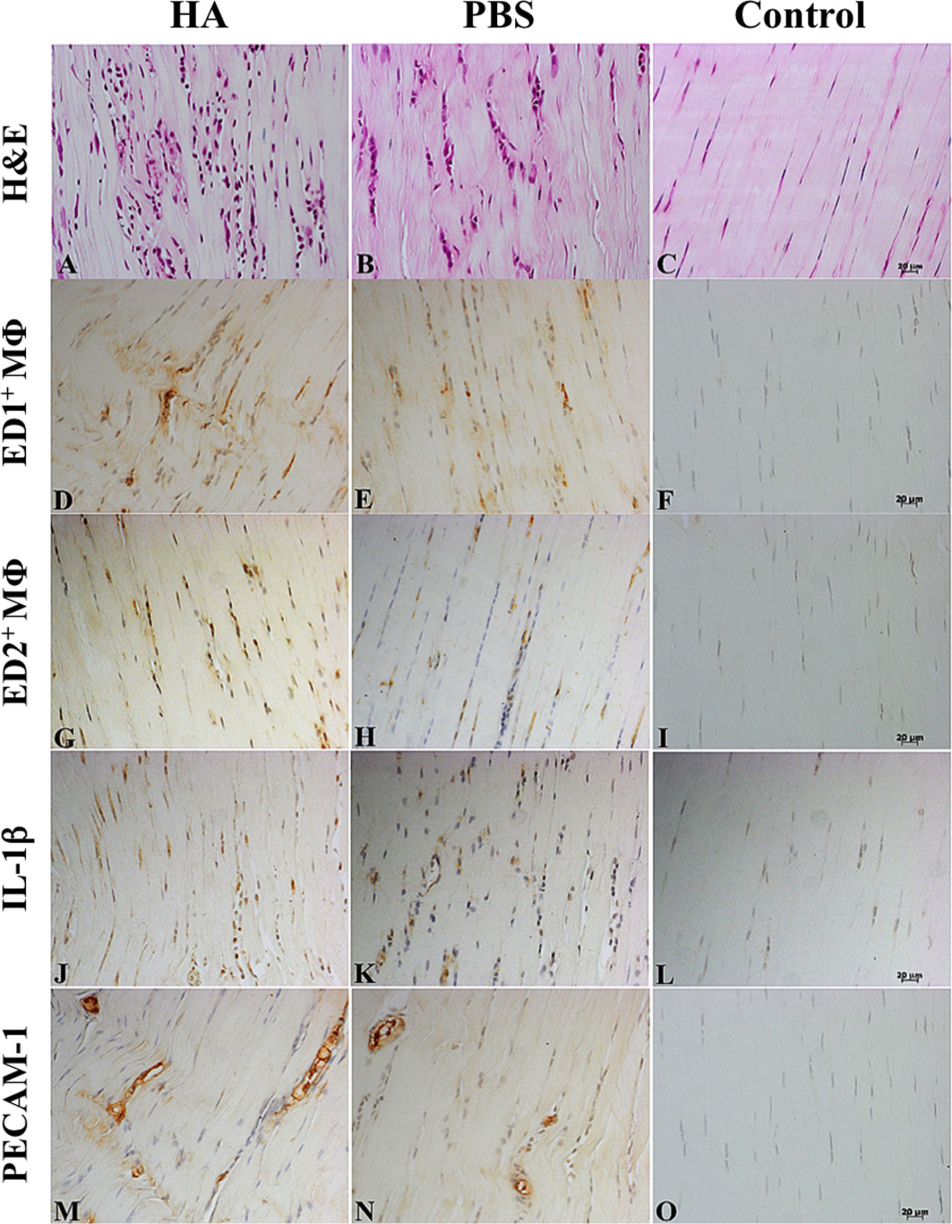
Immunohistochemistry images of histopathological changes after intratendinous injections. Rat Achilles tendons stained with hematoxylin and eosin (H&E) (A-C), ED1^+^ macrophages (D-F), ED2^+^macrophages (G-I), IL-1β (J-L), and platelet endothelial cell adhesion molecule (PECAM-1) (M-O) on *day 7* after an intratendinous injection ofhyaluronate (HA), phosphate buffered saline (PBS), or neither (Control: needle punctures only), respectively (from left to right). Magnification: 200x; bar = 20μm.

**Fig 3:**
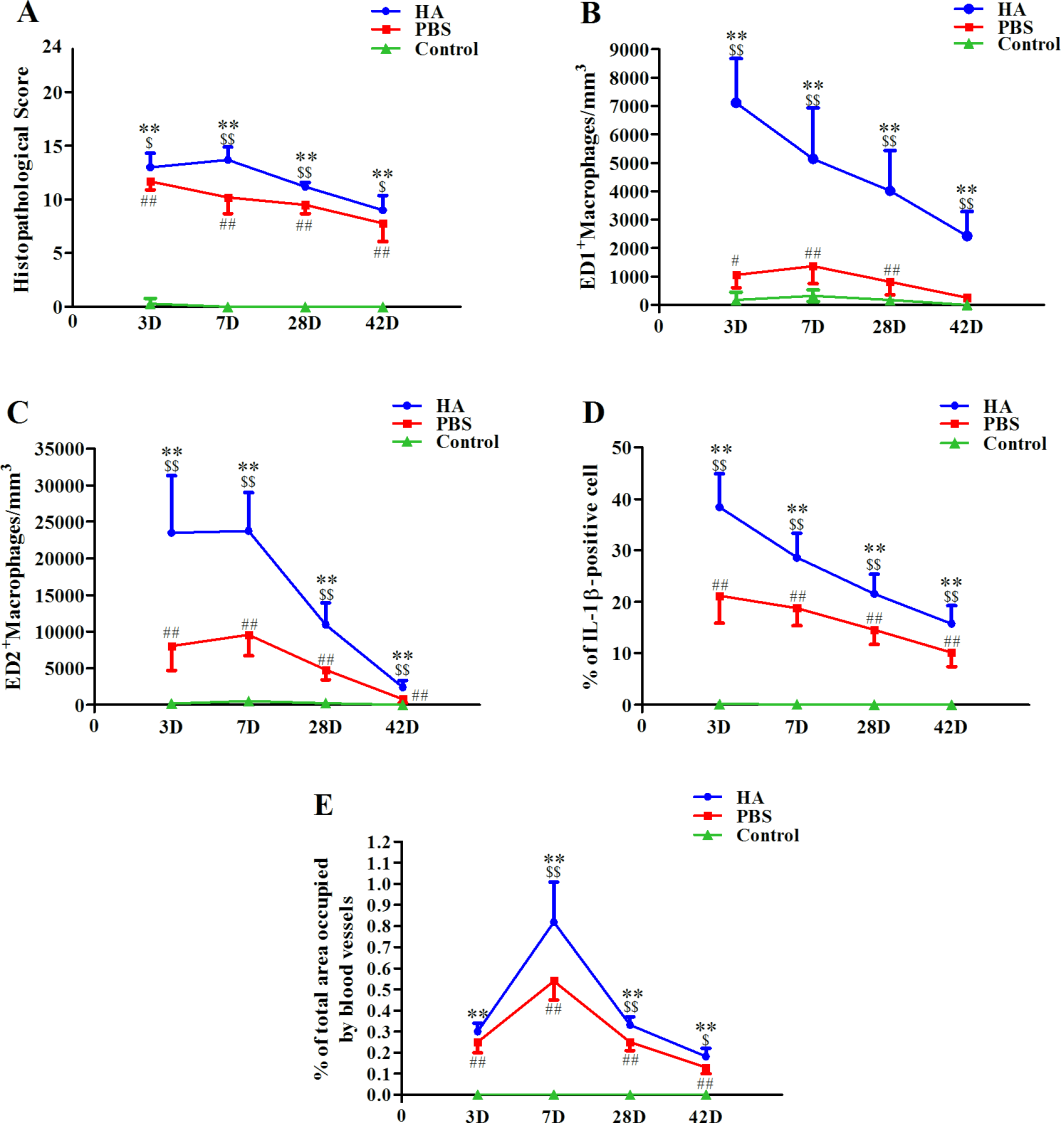
Dynamic expressions of acute inflammation characteristics after intratendinous injections. Histopathological score (A), ED1+ macrophage density (B), ED2+ macrophage density (C), the proportion of IL-1β+ cells (D), and the proportion of neovascularization areas (E) after an intratendinous injection with hyaluronate (HA), phosphate buffered saline (PBS), or neither (Control: needle punctures only). */# compare with Control group; $ compare with PBS group; $/# p<0.05; **/$ $/## p<0.01.

Tendons given an intratendinous injection of either HA or PBS showed acute tendon injury compared with the Control group (Figs 2A–2C and 3A). In the HA group, the histopathological score peaked on post-injection *day 3*, maintained that peak until *day 7*, and then significantly declined until *day 42*. In the PBS group, the histopathological score peaked on post-injection *day 3* and then declined until *day 42* (Fig 3A). Compared with the PBS group, the HA group showed significantly (*p*< 0.05 for all) more severe histopathological changes from *day 3* until *day 42*. The HA and PBS groups showed significantly (*p*< 0.01 for all) more severe injury than did the Control group throughout the study (Fig 3A; S1 Table). The interobserver agreement for the histopathological score was 0.88.

## ED1^+^ and ED2^+^ macrophage density and IL-1β expression

Tendons given an intratendinous injection of either HA or PBS showed acute inflammation (Fig 2D–2L). In the HA group, ED1^+^ and ED2^+^MΦ densities both peaked on *day 3* (7112 ± 1540/mm^3^ and 23475 ± 7825/mm^3^, respectively), ED1^+^MΦ densities gradually declined until *day 42* (Fig 3B; S2 Table), and ED2^+^MΦ densities significantly declined after *day 7* (Fig 3C; S3 Table). In the PBS group, ED1^+^ and ED2^+^MΦ densities peaked on *day 7* and then gradually declined until *day 42*. Compared with the PBS and Control groups, the ED1^+^ and ED2^+^MΦ densities in the HA group were significantly (*p*< 0.01 for all) higher at each time-point. The PBS group showed significantly (*p*< 0.01 for all) higher ED1^+^ and ED2^+^MΦ densities than did the Control group, except for ED1^+^MΦ density on *day 42* (Fig 3B and 3C; S1 and S2 Tables). The IL-1β expression peaked on *day 3* in both the HA and PBS groups, and then gradually decreased. In the HA group, the IL-1β expression was significantly (*p*< 0.01 for all) higher than in the PBS group throughout the study. Compared with the Control group, both the HA and the PBS groups consistently showed significantly (*p*< 0.01 for all) higher IL-1β expression at all time-points (Fig 3D; S4 Table).

### Neovascularization

Neovascularization is clearly depicted both in the HA group and in the PBS group (Fig 2M–2O). The neovascularization area peaked on *day 7* (0.82 ± 0.19% vs. 0.54 ± 0.09%), and then gradually decreased until *day 42*. The proportion of areas in which angiogenesis occurredin the HA group was significantly (*p*< 0.05 for all) higher than in the PBS group from *day 7* to *day 42* (Fig 3E; S5 Table).

## Discussion

This is the first study that examines the acute inflammatory responses after US-guided intratendinous injections of HA. Each Achilles tendon in the HA and PBS groups showed substantial and significant histopathological changes compatible with acute tendon injury, corresponding inflammatory ED1^+^ and ED2^+^MΦ infiltration, IL-1β expression, and neovascularization compared with the Control group. All of these changes were significantly more severe in the HA group than in the PBS group at each time-point.

The infiltration of inflammatory neutrophils and macrophages is part of a sequence of inflammatory cell accumulation involved in tendon repair[21]. The function of MΦs in wound healing is less conflicting than that of neutrophils. MΦs secrete several kinds of cytokines that regulate angiogenesis, chemotaxis[26], fibroblast proliferation[27], extracelluar matrix synthesis, and remodeling[28]. Different subtypes of MΦs might have complementary functions during tissue inflammation and healing. For instance, ED1^+^MΦs are phagocytic cells present primarily in circulation, and they migrate into injured skeletal muscle to remove necrotic debris[25]. ED2^+^MΦs are endogenous cells and are associated with the regeneration stage of muscle injury[29]. Acute tendon injury first shows a greater accumulation of ED1^+^MΦs, which is followed by increased ED2^+^MΦ infiltration in a collagenase-induced model[21, 23]. The accumulation of ED2^+^MΦs tended to increase until post-injection *day 28*. Therefore, it is believed that ED1^+^MΦs rule tendon catabolism and that ED2^+^MΦs rule tendon anabolism[21]. In our study, the HA and PBS groups showed relatively higher MΦ density until post-injection *day 7*, after which the density gradually decreased until *day 42*. This trend of MΦ accumulation was similar to that in the acute injection-induced inflammation model reported by Chbinou et al.[23]. Throughout our study, both ED1^+^ and ED2^+^MΦ densities were significantly higher in the HA group than in the PBS group, especially on *day*s *3* and *7*. The results indicated that intratendinous injections of HA induced a more severe inflammatory reaction that corresponded to a higher histopathological score, higher IL-1β expression, and more neovascularization in the HA group. Furthermore, a dramatically high level of IL-1β was revealed in the early stage of tendon injury and was regarded as necessary for subsequent tendon healing[22, 30]. MΦs are also known to secrete IL-1 and tumor necrosis factor, both of which inhibit fibroblast proliferation and degrade extracellular matrix[31]. Such exogenous IL-1β also induces tenocytes to secrete endogenous IL-1β through an autocrine or paracrine action. Therefore, IL-1β expression substantially increased after intratendinous injections of HA and of PBS, and the trend of IL-1β expression was compatible with the trend of ED1^+^ and ED2^+^MΦ density. The sharp pain caused by an intratendinous injection of HA might be the result of acute inflammation. The exact mechanism of an intratendinous HA injection that leads to acute tendon injury is still unknown. There are two possible mechanisms. One is the volume effect: an intratendinous injection expands the tendon and induces tendon lamination. Another is the viscosity of HA, which is much higher than that of PBS[15]. Our findings that both the HA and PBS groups showed acute tendon injury and that the injury in HA group was more severe correspond to the possible mechanisms. Our results also explain why the studies evaluating the effects of HA in treating tendinopathy adopted paratendinous rather than intratendinous injections[9, 11, 12, 15, 17, 18]. Therefore, US guidance, and any other method that increases the accuracy of the injection and eliminates intratendinous injections, should be promoted in clinical practice.

Neovascularization, which occurs during acute tendon injury[23] and tendinopathy[32], is essential for tendon healing. Initially, the injured tendon is filled by a scaffold composed of exudated fibronectin and fibrin, which allows for the sequential migration of various cells and stimulates vascular ingrowth. A continuing source of growth factors released from MΦs stimulates fibroblast proliferation and angiogenesis. The induction of angiogenesis might be initiated by basic fibroblast growth factor during the first 3 days of wound healing. Later, vascular endothelial growth factor follows in the angiogenesis that occurs during the formation of granulation tissue on *days 4–7*[26]. Chbinou et al.[23]reported that, in a collagenase-induced inflammation model, angiogenesis peaked in the paratendon on *day 7* and in the endotendon on *day 14*. In our study, angiogenesis in the endotendon peaked on *day 7* in both the HA and the PBS groups. The dynamic expression of angiogenesis indicated that the intratendinous injections of HA and PBS induced acute tendon injury and initiated the process of tendon healing. A possible explanation for the earlier peak time of angiogenesis in our study might be that the rats in our study had less damage than did those in Chbinou et al[23]. Otherwise, the earlier peak time of angiogenesis in our study might be related to less damage than in the collagenase-injection model.

Interestingly, rats intratendinously injected with normal saline or PBS were once used as controls because it was believed that they had no post- injection inflammation[21, 33]. However, Andersson et al.[34]reported that an intratendinous injection of saline induced hypercellularity to almost the same degree as did an intratendinous injection of substance P. Cellular proliferation is considered an adaptive response because of the increased local tissue pressure or because of a cascade after the possible local release of substance P or inflammatory cytokines caused by an intratendinous injection of saline. In our study, the PBS group showed acute tendon injury and inflammatory responses, including hypercellularity, with other histopathological changes, sequential accumulation of ED1^+^ and ED2^+^MΦs, identifiable IL-1β expression, and angiogenesis, even though the changes were less severe than those in the HA group. The dramatic differences after an intratendinous injection of PBS might be related to the volume effect. A higher volume of PBS was used in our study (0.1 ml) and in Andersson et al.[34] (1 ml) than in Marsolais et al.[21](20 μl) and Lui et al.[33](30 μl). The high volume-image guided injection effect is one possible clinical factor in tendinopathy treatment[35]. Another is the accuracy of the injection. Conventionally, an intratendinous injection is blindly and percutaneously done[21, 33]. Recently, US-guided injections are favored to confirm the accuracy of the injection[34, 36], as we did in the present study. Therefore, a large-volume intratendinous injection should be used only after judicious consideration because it might cause acute inflammation.

Our study has some limitations. First, we use healthy rather than tendinopathic tendons in our study. However, the current concept of pathogenesis in tendinopathy is that inflammation and degeneration synergistically contribute to tendinopathy[32]. Consequently, the current histopathological scoring system on tendinopathy is substantially similar to that used for acute tendinitis; neovascularization is also a hallmark of either tendinopathy or acute tendinitis. The similar assessment methodologies would confound the evaluation of an acute inflammation episode after intratendinous injections in a tendinopathy (i.e., chronic inflammation) animal model. Our aim was to evaluate the acute inflammation process after intratendinous injections, not the therapeutic effect of HA in tendinopathy. Therefore, rats with healthy tendons were used to avoid unnecessary bias, and the animal model clearly and adequately revealed cellular responses after an intratendinous injection. Second, the sample size in each group was small. However, the number of experimental and control rats was acceptable for an animal study and adequately depicted the differences between groups.

## Conclusion

We conclude that an intratendinous injection of either HA or PBS induces acute tendon injury and inflammatory reactions: histopathological changes, ED1^+^ and ED2^+^ MΦs accumulation, IL-1β expression, and neovascularization. The injury induced by the intratendinous injection of HA was more severe than that induced by PBS. Our findings confirm our hypothesis that an intratendinous injection of HA induces acute inflammation, worse histopathological results, more MΦs infiltration, and more angiogenesis than induced by an intratendinous injection of PBS. Our findings tell us to avoid intratendinous injections of HA because they cause acute inflammation.

## Supporting Information

S1 Table Results of histopathological scoring in Achilles tendons after an intratendinous injection. (DOCX)

S2 Table Results of ED1^+^ macrophage density in Achilles tendons after an intratendinous injection. (DOCX)

S3 Table Results of ED2^+^ macrophage density in Achilles tendons after an intratendinous injection. (DOCX)

S4 Table Results of the proportion of IL-1β^+^ cells in Achilles tendons after an intratendinous injection. (DOCX)

S5 Table Results of the proportion of neovascularization in Achilles tendons after an intratendinous injection. (DOCX)
