# Supplementary material for: Intratendinous Injection of Hyaluronate Induces Acute Inflammation: A Possible Detrimental Effect
Source: PLoS One. 2016 May 13;11(5):e0155424. doi: 10.1371/journal.pone.0155424 (PMC4866702; doi:10.1371/journal.pone.0155424)
Supplement: S1 Table — (DOCX) [file pone.0155424.s001.docx]

**S1 Table.** **Results of histopathological score in Achilles tendons after an intratendinous injection.**

| **Histopathological** **Score** | ***Day 3*** | ***Day 7*** | ***Day 28*** | ***Day 42*** |
| --- | --- | --- | --- | --- |
| **HA** | 12.6 ± 0.8 | 12.6 ± 1.1 | 9.3 ± 0.7 | 8.3 ± 1.1 |
| **PBS** | 11.5 ± 0.8 | 10.3 ± 1.6 | 7.4 ± 0.7 | 7.0 ± 0.7 |
| **Control** | 0.3 ± 0.5 | 0 ± 0 | 0 ± 0 | 0 ± 0 |
| ***P-value*** |  |  |  |  |
| Within groups | < 0.001 | < 0.001 | < 0.001 | < 0.001 |
| HA vs. PBS | 0.021 | 0.005 | 0.001 | 0.026 |
| HA vs. control | 0.002 | 0.001 | 0.001 | 0.001 |
| PBS vs. control | 0.002 | 0.001 | 0.001 | 0.001 |

HA: hyaluronate; PBS: phosphate buffered saline.

The differences in all groups were analyzed using the Kruskal-Wallis test and the post-hoc test was done using the Mann-Whitney U test.
