# Supplementary material for: Intratendinous Injection of Hyaluronate Induces Acute Inflammation: A Possible Detrimental Effect
Source: PLoS One. 2016 May 13;11(5):e0155424. doi: 10.1371/journal.pone.0155424 (PMC4866702; doi:10.1371/journal.pone.0155424)
Supplement: S3 Table — (DOCX) [file pone.0155424.s003.docx]

**S3 Table.** **Results of ED2^+^ macrophage density in Achilles tendons after an intratendinous injection.**

| **ED2^+^ MΦ/mm^3^** | ***Day 3*** | ***Day 7*** | ***Day 28*** | ***Day 42*** |
| --- | --- | --- | --- | --- |
| **HA** | 23482 ± 7817 | 23750 ± 5242 | 10938 ± 2993 | 2396 ± 938 |
| **PBS** | 8036 ± 3328 | 9554 ± 2830 | 4777 ± 1390 | 766 ± 509 |
| **Control** | 175 ± 275 | 500 ± 275 | 225 ± 275 | 0 ± 0 |
| ***P-value*** |  |  |  |  |
| Within groups | < 0.001 | < 0.001 | < 0.001 | < 0.001 |
| HA vs. PBS | 0.001 | 0.001 | 0.001 | 0.003 |
| HA vs. control | 0.002 | 0.002 | 0.002 | 0.001 |
| PBS vs. control | 0.002 | 0.002 | 0.002 | 0.001 |

MΦ: macrophages; HA: hyaluronate; PBS: phosphate buffered saline.

The differences in all groups were analyzed using the Kruskal-Wallis test and the post-hoc test was done using the Mann-Whitney U test.
