# Supplementary material for: Intratendinous Injection of Hyaluronate Induces Acute Inflammation: A Possible Detrimental Effect
Source: PLoS One. 2016 May 13;11(5):e0155424. doi: 10.1371/journal.pone.0155424 (PMC4866702; doi:10.1371/journal.pone.0155424)
Supplement: S4 Table — (DOCX) [file pone.0155424.s004.docx]

**S4 Table.** **Results of the proportion of IL-1β^+^ cells in Achilles tendons after an intratendinous injection.**

| **IL-1β-positive cells** (**%)** | ***Day 3*** | ***Day 7*** | ***Day 28*** | ***Day 42*** |
| --- | --- | --- | --- | --- |
| **HA** | 38.42 ± 6.47 | 28.59 ± 4.75 | 21.57 ± 3.85 | 15.78 ± 3.48 |
| **PBS** | 21.23 ± 5.37 | 18.78 ± 3.38 | 14.62 ± 2.90 | 10.16 ± 2.72 |
| **Control** | 0.18 ± 0.11 | 0.11 ± 0.73 | 0.02 ± 0.03 | 0.00 ± 0.01 |
| ***P-value*** |  |  |  |  |
| Within groups | < 0.001 | < 0.001 | < 0.001 | < 0.001 |
| HA vs. PBS | 0.001 | 0.001 | 0.006 | 0.006 |
| HA vs. control | 0.002 | 0.002 | 0.002 | 0.002 |
| PBS vs. control | 0.002 | 0.002 | 0.002 | 0.002 |

HA: hyaluronate; PBS: phosphate buffered saline.

The differences in all groups were analyzed using the Kruskal-Wallis test and the post-hoc test was done using the Mann-Whitney U test.
