# Supplementary material for: Intratendinous Injection of Hyaluronate Induces Acute Inflammation: A Possible Detrimental Effect
Source: PLoS One. 2016 May 13;11(5):e0155424. doi: 10.1371/journal.pone.0155424 (PMC4866702; doi:10.1371/journal.pone.0155424)
Supplement: S5 Table — (DOCX) [file pone.0155424.s005.docx]

**S5 Table.** **Results of the proportion of neovascularization in Achilles tendons after an intratendinous injection.**

| **Neovascularization**  **area** (**%)** | ***Day 3*** | ***Day 7*** | ***Day 28*** | ***Day 42*** |
| --- | --- | --- | --- | --- |
| **HA** | 0.30 ± 0.04 | 0.82 ± 0.19 | 0.33 ± 0.04 | 0.18 ± 0.04 |
| **PBS** | 0.25 ± 0.05 | 0.54 ± 0.09 | 0.25 ± 0.04 | 0.13 ± 0.03 |
| **Control** | 0.00 ± 0.00 | 0.00 ± 0.00 | 0.00 ± 0.00 | 0.00 ± 0.00 |
| ***P-value*** |  |  |  |  |
| Within groups | 0.001 | < 0.001 | < 0.001 | 0.001 |
| HA vs. PBS | *0.052* | 0.003 | 0.006 | 0.046 |
| HA vs. control | 0.001 | 0.001 | 0.001 | 0.001 |
| PBS vs. control | 0.001 | 0.001 | 0.001 | 0.001 |

HA: hyaluronate; PBS: phosphate buffered saline.

The differences in all groups were analyzed using the Kruskal-Wallis test and the post-hoc test was done using the Mann-Whitney U test.
